# Supplementary material for: Knowledge and awareness-based survey of COVID-19 within the eye care profession in Nepal: Misinformation is hiding the truth
Source: PLoS One. 2021 Jul 21;16(7):e0254761. doi: 10.1371/journal.pone.0254761 (PMC8294537; doi:10.1371/journal.pone.0254761)
Supplement: S1 Data — (DOCX) [file pone.0254761.s001.docx]

**1. What does the object in the picture look like?**

**A. SARS-CoV-2**

**B. COVID-19**

C. Sponge Ball

**D. Both A & B are correct**


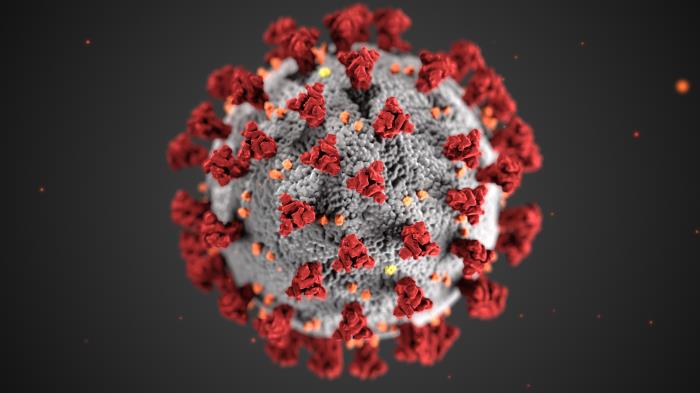


*This question was removed after being asked as it was deemed confusing so was not included in analysis.*

**2. What are the symptoms of coronavirus if it gets into human body?**

A. Asymptomatic or mild cough

B. Rise in body temperature

C. Shortness of breath

D. Severe cough with pneumonia

**E. All the above**

F. Don’t know

**3. What does PPE stand for?**

**A. Personal Protective Equipment**

B. Personal Protective Essentials

C. Don’t know

**4. Who is most likely to get infected from coronavirus? (Two responses allowed)**

A. Old age people

B. Immune compromised people

C. Everyone

D. Not children

E. Don't know

F. Mixed responses

**5. How does coronavirus spread? (2 response allowed)**

A. Respiratory droplets from infected person while coughing or sneezing.

B. Close contact with infected person or the carrier.

C. Going into public gatherings.

D. It is air borne and can infected if the air is contaminated with COVID19 pathogen.

F. All the above G. Mixed responses (ABD)

**6. What does RDT stand for?**

A. Rapid Disease Test

**B. Rapid Diagnostic Test**

C. Repeat Diagnostic Test

D. Don’t know

**7. What is the confirmatory test for SARS –CoV-2?**

A. DNA sequencing

B. PCR

**C. RT-PCR**

D. Viral Culture

E. Don’t know

**8. What would you do if you find someone coughing with high fever in your locality?**

A. Inform the health department by calling the toll-free number

B. Ask the patient to seek medical care soon and make sure they isolate themselves from others

C. Do nothing but self-isolate

D. Depends on the person who has symptoms

E. Don’t know

**9. What are the preventive measures the WHO has imposed to minimise coronavirus spread? (More than one response allowed)**

A. Wash Hands frequently with alcohol-based disinfectant or soap water

B. Maintain social distance C. Cover mouth, nose while sneezing and coughing

D. Wear protective, goggles, face mask, avoid touching face without hand wash

E. All the above

F. Don’t know

G. Mixed responses (A, B, D)

**10. What do you think would be best to reduce the mortality rate caused by COVID-19?**

A. Lockdown and social distancing

B. No roaming around, stay away from people having flu

C. Early consultation if any symptoms noticed D. Mandatory COVID19 test if came across any infected patient

E. Don’t know

**11. Consuming hot fluids, garlic/ginger mix, Vitamin C soup etc. regularly kills the virus. Do you agree?**

A. Yes **B. No** C. Don’t know

**12. How long does it take the virus to die from the contaminated surface?**

A. 4-5 days in plastic, paper, glass, and wood

B. Up to 2 days in any Metal surface

C. 12 hours in gloves and 2-3 days in masks

**D. All the above**

E. Don’t know

**13. How frequently do you wash or dispose of your face mask?**

A. I wash it daily

B. I don’t use mask C. I use new one daily

D. I wash it daily in case I don’t forget

E. Once every two days.

*(Both A and C were aggregated to one option)*

**14. What is the appropriate time for handwashing?**

A. 1 min

B. 40 Sec

**C. 20 Sec**

D. saying (not singing) happy birthday

E. Both A & D

**15. How often do you touch your face without washing or sanitising your hand?**

A. I don't do it without washing my hands afterwards.

B. I don't have the habit of touching face.

C. I don't remember if I touch my face.

D.I use my non dominant hand in doing most tasks. I touch my face with my dominant hand.

E. Less often, mostly when I am consciously doing something else.

**16. Which are the most effective disinfectants for use in ophthalmic clinics? (two responses allowed)**

A. Absolute alcohol **B. 70% ethanol**  C. Isopropyl alcohol D. 0.5% sodium hypochlorite

E. All the Above F. B and D are effective

G. Don’t know

**17. How significant is the knowledge of the Coronavirus for eye care practitioners?**

A. Very significant B. Somewhat significant

C. Don’t idea

**18. Which one among these complications of the eye is related to COVID-19?**

**A. Conjunctivitis**

B. Epiphora

C. Glaucoma

D. Keratitis

E. Don’t know

**19. How safe is the clinic you work in terms of patients' exposure, having symptoms of coronavirus, and red eye? (two responses allowed)**

A. I have a PPE and my Slit lamp has shield

B. I use gloves and mask also sanitize my hand

C. Slit lamp has shield and I wear mask and gloves

D. My clinic has considered all the safety measures to fight this pandemic

**20. How are you helping the community combat this pandemic?**

A. My clinic entertains only emergency care with high protection

B. I am in self-isolation

C. I follow the instructions given by WHO in restricting the disease spread.

D. My clinic is closed temporarily

E. I do voluntary support to in community with self-protective measures.

**21. What should elderly people do during the period of lockdown?**

A. I don't have elderly people in my home

B. Stay home

C. No change to usual activities

D. We should take them for a walk outside daily as before

E. Don’t know

**22. What is an ocular emergency within the context of COVID-19?**

A. Sudden loss of vision

B. Painful Red eye

C. Ocular trauma

D. Blurred vision

**E. All except blurred vision**

**23. Being an eye care practitioner, what do you think is wise to do during lockdown?**

A. Online training/webinar, new skills B. Read books and research papers

C. Only emergency cases to be seen at clinic/opd D. Social distancing E Social Activity

F Shut down the clinic and stay at home

G. Follow the WHO guideline

*(Free-text question, with answers grouped into categories)*

**24. Avoid investigations in ophthalmology such as tonometry, OCT, refraction, etc. until this pandemic resolve. Do you agree?**

A. Yes

B. No*

*why?

- Emergency cases only
- Perform with disease safety measure to aid in diagnosis
- Should be done if patient has come to out-patient department

*(Free text option for reasoning if the answer given is No)*

**25. Eye care practitioners should use PPE during this pandemic of COVID-19, to what extent do you agree?**

A. Agree

B. Strongly agree

C. Neutral

D. Strongly disagree
