# Supplementary material for: Knowledge and awareness-based survey of COVID-19 within the eye care profession in Nepal: Misinformation is hiding the truth
Source: PLoS One. 2021 Jul 21;16(7):e0254761. doi: 10.1371/journal.pone.0254761 (PMC8294537; doi:10.1371/journal.pone.0254761)
Supplement: Attachment — Submitted filename: Response to Reviewers.docx [file pone.0254761.s002.docx]

**Editorial comments:**

1) Please ensure that your manuscript meets PLOS ONE's style requirements, including those for file naming. The PLOS ONE style templates can be found at

<https://journals.plos.org/plosone/s/file?id=wjVg/PLOSOne_formatting_sample_main_body.pdf> and

<https://journals.plos.org/plosone/s/file?id=ba62/PLOSOne_formatting_sample_title_authors_affiliations.pdf>

Thank you for reviewing the paper. We have edited our manuscript as per the guidelines of PLOS ONE.

2)  Please provide additional details regarding participant consent. In the ethics statement in the Methods and online submission information, please ensure that you have specified (1) whether consent was informed and (2) what type you obtained (for instance, written or verbal, and if verbal, how it was documented and witnessed). If your study included minors, state whether you obtained consent from parents or guardians. If the need for consent was waived by the ethics committee, please include this information.

Thank you for this comment. We have edited and clarified this in the method. The relevant section now reads as follows:

A mandatory informed written consent was taken digitally prior to participation. There were no patients or minors involved in this study.

3) Thank you for including your ethics statement:  "Ethical permission was obtained".

Please amend your current ethics statement to include the full name of the ethics committee/institutional review board(s) that approved your specific study.

Once you have amended this/these statement(s) in the Methods section of the manuscript, please add the same text to the “Ethics Statement” field of the submission form (via “Edit Submission”).

For additional information about PLOS ONE ethical requirements for human subjects research, please refer to <http://journals.plos.org/plosone/s/submission-guidelines#loc-human-subjects-research>.

We have now edited this as per the PLOS ONE requirement, thank you. The updated section now reads as follows:

Ethical permission was obtained from Ethical Review Board (ERB) of Sagarmatha Choudhary Eye Hospital, Ref 49-2077/078, dated 1^st^ of March 2020.

4) Thank you for stating the following in your Competing Interests section:

[nil].

Please complete your Competing Interests on the online submission form to state any Competing Interests. If you have no competing interests, please state "The authors have declared that no competing interests exist.", as detailed online in our guide for authors at http://journals.plos.org/plosone/s/submit-now

 This information should be included in your cover letter; we will change the online submission form on your behalf.

Thank you, it has been included in the cover letter. The updated Competing Interests section now reads:

"The authors have declared that no competing interests exist."

Please know it is PLOS ONE policy for corresponding authors to declare, on behalf of all authors, all potential competing interests for the purposes of transparency. PLOS defines a competing interest as anything that interferes with, or could reasonably be perceived as interfering with, the full and objective presentation, peer review, editorial decision-making, or publication of research or non-research articles submitted to one of the journals. Competing interests can be financial or non-financial, professional, or personal. Competing interests can arise in relationship to an organization or another person. Please follow this link to our website for more details on competing interests: http://journals.plos.org/plosone/s/competing-interests

5) We note that you have indicated that data from this study are available upon request. PLOS only allows data to be available upon request if there are legal or ethical restrictions on sharing data publicly. For information on unacceptable data access restrictions, please see <http://journals.plos.org/plosone/s/data-availability#loc-unacceptable-data-access-restrictions>.

In your revised cover letter, please address the following prompts:

a) If there are ethical or legal restrictions on sharing a de-identified data set, please explain them in detail (e.g., data contain potentially identifying or sensitive patient information) and who has imposed them (e.g., an ethics committee). Please also provide contact information for a data access committee, ethics committee, or other institutional body to which data requests may be sent.

There are no restrictions as such, data is made available online. Thank you. This has been explained within the cover letter and we have made our data available through an online repository (Dryad).

b) If there are no restrictions, please upload the minimal anonymized data set necessary to replicate your study findings as either Supporting Information files or to a stable, public repository and provide us with the relevant URLs, DOIs, or accession numbers. Please see <http://www.bmj.com/content/340/bmj.c181.long> for guidelines on how to de-identify and prepare clinical data for publication. For a list of acceptable repositories, please see <http://journals.plos.org/plosone/s/data-availability#loc-recommended-repositories>.

We will update your Data Availability statement on your behalf to reflect the information you provide.

Data is made available to the online repository system, Dryad, and can be viewed with the following DOI: <https://doi.org/10.5061/dryad.bcc2fqzbx>

6) Please amend either the title on the online submission form (via Edit Submission) or the title in the manuscript so that they are identical.

This has been edited on the online submission, thank you.

7)  We noticed you have some minor occurrence of overlapping text with the following previous publication(s), which needs to be addressed:

<https://onlinelibrary.wiley.com/doi/abs/10.1002/jmv.25725>
<https://www.cehjournal.org/article/understanding-covid-19-and-the-virus-that-causes-it/>

In your revision ensure you cite all your sources (including your own works), and quote or rephrase any duplicated text outside the methods section. Further consideration is dependent on these concerns being addressed.

Thank you for the suggestion, it is paraphrased and cited now. The relevant sections now read as follows:

The Center for Disease Control and prevention, China, extracted and identified the causative virus as a unique coronavirus. Following this, the World Health Organization (WHO) named this new pathogen the 2019-novel-Coronavirus, [3] and suggested on 25th Dec 2019 that the virus is less contagious to healthcare professionals compared to other recent coronaviruses responsible for severe acute respiratory syndrome (SARS) and the Middle East respiratory syndrome (MERS). [4] …

…The majority of the participants knew that conjunctivitis was the most common ocular manifestation of COVID-19, which is reassuring and has not changed over some time. Different types of conjunctivitis and keratoconjuctivitis are the most frequently reported ocular manifestations, with one case-report of a patient demonstrating retinal changes detected by ocular coherence tomography.[31-33]

**Comments to the Author**
**Reviewer #1:** 
1. The paper is about a contemporary issue in public health, it is clearly written, and the topic is of high importance. The abstract is clear. Title is ambiguous.

Thank you very much for reviewing our manuscript and for providing valuable feedback. Thank you for appreciating the importance of this topic. Regarding the title, we have amended the title on the online system so that there is no confusion.

2. The introduction should give background to the choice of study participants for the study, should demonstrate gap in knowledge, justification for the study and show relevance of the research to policy and practice. Why eye care practitioners? Why is the study relevant? Will it help to better control the emergency? All these did not appear clearly in the introduction.

Thank you for this comment. On a pilot online questionnaire of the same questions among 10 participants before beginning the study, the knowledge and awareness seemed poor. We therefore felt there was a need to conduct a larger, more thorough study to check if the perception was the same or different across the entire nation. Eye care practitioners are at close contact with patients and there is a high risk of transmitting COVID-19 virus through aerosols between patients and eye care personnel.

Dr Li, an ophthalmologist from Wuhan who unfortunately died from COVID-19 early during the pandemic, had said COVID-19 needs to be taken seriously by eye care workers in order to protect themselves. References to this have now been added to the introduction.

If gaps in knowledge were found, then these could be addressed through targeted education / training which helps reduce the risk to healthcare workers and their patients and promote good infection control practices.

The introduction has now been updated to reflect these points and now reads as follows:

During this period, there have been many rumors and misinformation on COVID-19 emerging, which might mislead ophthalmic personnel.

Dr Li Wenliang from Wuhan, who died from COVID-19, was an ophthalmologist who warned about the contagious nature of this virus and had asked eye care practitioners worldwide to take safety precautions, given the close proximity eye care practitioners are in with their patients. The aerosols generated from diseased patients attending for eye examinations have a higher tendency to infect eye care personnel if the appropriate protection is lacking. A pilot study of ours using the same study tool revealed poor knowledge and awareness within the eye care practitioners at our institution. The aim of this study was to review the knowledge and awareness of COVID-19 amongst eye care personnel in Nepal. If gaps in knowledge were found, then this could be addressed through targeted education and training, which will help reduce the risk to healthcare workers and promote good infection control practices.

3. The method: Authors should state who commissioned the study. What IRB granted approval, approval date and number (see line 88). It is important to state how the sample size for the study was determined, how instrument for data collection was checked for validity and reliability.

Thank you for these comments. As noted in the response to the Editor, we have updated the ethics statement within the methods section to include the details of the ERB, approval date and number. Please see the response above which includes the updated ethics statement.

Regarding the sample size, we have added the following text to the manuscript to address the logic behind how this was determined. This section now reads as follows:

The survey was sent to 600 of these clinicians working in Nepal. Participation was anonymous and voluntary. Representatives from a random selection of 12 of the 18 secondary or tertiary eye hospitals and clinics across the spectrum of health facilities within Nepal were contacted and asked to select 50 individuals randomly at their institution and contacts who were involved in eye care practice during COVID-19, who were then invited to take the survey after their agreement. This random sample of 600 ophthalmic clinicians (approximately 40% of eye care professionals within Nepal) was calculated to be representative of the staff involved in eye care, whilst being practical enough to implement, making use of established networks between eye care providers.

Regarding the validity, online forms could only be completed by those directly invited and values were entered directly by the individual. This has been clarified within the text and this section now reads as follows:

In order to ensure validity, online forms could only be completed by those directly invited and values were entered directly by the individual.

4. The choice of analytical approach is too basic. There is no explanation of the independent and dependent variables. There is need to conduct inferential statistics, compute mean scores, standard deviations and ratios to better explain the knowledge and awareness scores obtained.

Thank you for this observation. This study was performed as a survey of the views of ophthalmic professionals, and the results reported in the knowledge section represent the percentage of question that our sample correctly answered. Therefore, reporting the results in this way, it is not possible to do any further statistical analysis. We have, however, clarified the mean results of all participants, and given the standard deviation for these results. We have only reported on the mean overall results for the Knowledge component of the questionnaire. We have not reported the mean correct results for the Awareness section, as for these questions there is no correct response.

Regarding the comment about independent and dependent variables, in this survey we are not establishing cause and effect; as such we do not have any dependent or independent variables to define.

We have updated the results section of the text with the following, to include standard deviations as suggested:

The calculated mean knowledge performance score was 69.65% (SD ± 22.81), falling into the “satisfactory” category.

5. It is always better to avoid starting a sentence with a figure. See lines 124,128,136.

This has been corrected, thank you for alerting us to this.

6. The References are recent and relevant, but some are incomplete. Authors may want to update their references with related publications on the subject matter.

This has been updated now, thank you.

7. Recommendation for further study appeared in the conclusion, it may be better situated in the discussion section.

Shifted the recommendation section within the discussion, thank you.

**Reviewer #2:** 1. Financial disclosure and competing interests should be edited according to the journal’s instructions.

We have edited as per PLOS ONE requirements, thank you.

2. IRB’s full name and committee number should be mentioned.

We have now mentioned this within the method section, thank you.

3. Authors should include whether the inform consent was given orally or in a written form.

Clarified the same in the method section of the manuscript, thank you.

4. Authors should consider emphasizing the specific importance of PPE and supplementary measures (e.g. clear plastic boards placed on the slit lamp) utilization in the eye clinic.

We have revealed the importance of breath shields and other PPE for eye care practitioner in the discussion section, thank you.

5. Authors should update their data and cite contemporary studies regarding ocular transmission of SARA-CoV-2 and ocular manifestations. They should try and compare it to what was known in the time the questionnaires were filled and try to explain, in their opinion, if the participants would answer differently in light of the current data.

Thank you for this comment. We have updated this in the discussion section.

6. If available, it would be helpful to know additional background data of the participants (e.g. size of clinic, number of patients seen in a certain amount of time, etc.).

Thank you for this suggestion. We have added this to the results section. The following sentence has been added:

These eye health personnel were from a diverse range of health facilities, from small single-practitioner clinics to stand-alone tertiary referral eye hospitals.

7. Authors should mention and regard the fact that the majority of participants were quite of a homogenous group and of relatively young age. They should elaborate on it in their discussion.

Thank you for raising this point. This is now explained within the discussion and currently reads as:

Within Nepal, the majority of eye health personnel fall within the 25-35 age bracket. Although the proportion of males and females within the eye health workforce nationally is unknown, a recent study from the Eastern Region of Nepal reported that approximately 55% of general health practitioners were female. [20] Within our study, the age range of our respondents was as expected, although with 55% of respondents being male, women may be slightly under-represented within this sample if the gender distribution for the eye health workforce is similar to that of general health practitioners; male participants may have been, for example, more willing to complete the online survey for cultural reasons.

8. In continuum to the previous comment, authors should try and explain their results in light of the cultural and occupational background of the participants.

Thank you for this comment. We have addressed this within our response to point No. 7 above.

**Reviewer #3:** accepted with no comments. the manuscript is well written. The manuscript describe a technically sound piece of scientific research with data that supports the conclusions. the manuscript discussed an important issue and it seems quite interesting that most of the participants are not aware enough about this pandemic.

Thank you for your review.

6. PLOS authors have the option to publish the peer review history of their article ([what does this mean?](https://journals.plos.org/plosone/s/editorial-and-peer-review-process#loc-peer-review-history)). If published, this will include your full peer review and any attached files.


If you choose “no”, your identity will remain anonymous but your review may still be made public.


**Do you want your identity to be public for this peer review?** For information about this choice, including consent withdrawal, please see our [Privacy Policy](https://www.plos.org/privacy-policy).

Reviewer #1: No

Reviewer #2: No

Reviewer #3: No

[NOTE: If reviewer comments were submitted as an attachment file, they will be attached to this email and accessible via the submission site. Please log into your account, locate the manuscript record, and check for the action link "View Attachments". If this link does not appear, there are no attachment files.]

While revising your submission, please upload your figure files to the Preflight Analysis and Conversion Engine (PACE) digital diagnostic tool, <https://pacev2.apexcovantage.com/>. PACE helps ensure that figures meet PLOS requirements. To use PACE, you must first register as a user. Registration is free. Then, login and navigate to the UPLOAD tab, where you will find detailed instructions on how to use the tool. If you encounter any issues or have any questions when using PACE, please email PLOS at [figures@plos.org](mailto:figures@plos.org). Please note that Supporting Information files do not need this step.

|  |  |
| --- | --- |
| Online training/webinar, develop new skills | 16.45% |
| Read books and research papers | 8.71% |
| Only emergency cases to be seen at clinic/OPD | 12.9% |
| Social distancing | 11.29% |
| Continue social activity | 7.74% |
| Close the clinic and stay at home | 29.68% |
| Follow the WHO guidelines | 13.23% |
