# Supplementary material for: Knowledge and awareness-based survey of COVID-19 within the eye care profession in Nepal: Misinformation is hiding the truth
Source: PLoS One. 2021 Jul 21;16(7):e0254761. doi: 10.1371/journal.pone.0254761 (PMC8294537; doi:10.1371/journal.pone.0254761)
Supplement: Attachment — Submitted filename: Response to Reviewers R2.docx [file pone.0254761.s003.docx]

Dear Editor

Thank you for the further comments and questions. We have now addressed these as follows, with responses in green and any changes to the manuscript in blue. We feel that this has helped to further improve the quality of this manuscript.

Yours faithfully

Sandip Das Sanyam

**Journal Comments:**

Please review your reference list to ensure that it is complete and correct. If you have cited papers that have been retracted, please include the rationale for doing so in the manuscript text or remove these references and replace them with relevant current references. Any changes to the reference list should be mentioned in the rebuttal letter that accompanies your revised manuscript. If you need to cite a retracted article, indicate the article’s retracted status in the References list and also include a citation and full reference for the retraction notice.

Reference list is now updated, and URLs are checked, thank you.

**Additional Editor Comments (if provided):**

The authors have not addressed the analysis section of the review comments. both reviewer 1 and 2 expressed the need for the authors to do further analysis to express demographic, social and occupational differences in knowledge score. the authors response on this is not acceptable.

Thank you for this comment. We have now run further analysis on the knowledge results in terms of the background demographics (gender, age group and occupation). Fisher’s exact analysis does not show any significant difference for these demographic variables and the knowledge score obtained. We have therefore added the following sentence to the results section:

We found no evidence to suggest an association between demographic variables (specifically age, gender and occupation) and answering the knowledge questions correctly (data not shown).

We have also added the following to the methods section to describe the statistical testing performed:

Fisher’s exact testing was performed to look for significant associations between categorical variables.

it is also not clear whether the instrument for data collection was adapted, or how it was developed. If the latter is the case, how issues around reliability and validity of instrument was addressed.

Thank you for highlighting this. We have added the following now to the methods section to describe how the questionnaire was developed:

We used qualitative methods to generate the questions on knowledge and awareness. This has been shown to enrich the quality of questionnaire items and to improve the content validity of the questionnaire. Questions developed in this way derives item generation from the population of interest, rather than that of the researchers. Focus group discussion took place between eye care professionals, statisticians, and microbiologists, facilitated by the investigators.

**Note:**

Please be informed that we have also added one more author cum supervisor to the manuscript.
